# Revisiting a long‐overlooked skull: Implications for the distribution of *Dinodontosaurus brevirostris* (Kannemeyeriiformes) in the Brazilian Triassic

**DOI:** 10.1002/ar.70056

**Published:** 2025-09-29

**Authors:** Julia Lara Rodrigues de Souza, João Lucas Da Silva, Voltaire D. P. Neto, Arielli Fabrício Machado, Juan A. Escobar, Felipe L. Pinheiro

**Affiliations:** ^1^ Laboratório de Paleobiologia Universidade Federal Do Pampa (UNIPAMPA) São Gabriel Brazil; ^2^ Stephanie Pierce Lab, Museum of Comparative Zoology Harvard University Cambridge Massachusetts USA; ^3^ Programa de Pós‐graduação em Zoologia do Museu Nacional Universidade Federal do Rio de Janeiro (UFRJ) Rio de Janeiro Brazil; ^4^ Sección Paleontología de Vertebrados CONICET‐Museo Argentino de Ciencias Naturales “Bernardino Rivadavia” Buenos Aires Argentina; ^5^ Consejo Nacional de Investigaciones Científicas y Técnicas (CONICET) Buenos Aires Argentina

**Keywords:** Anomodontia, Dicynodontia, morphology, Santa Maria Formation, taxonomy

## Abstract

Dicynodonts (Anomodontia: Dicynodontia) were one of the main groups of terrestrial tetrapods in Permian and Triassic faunas. In Brazil, the genus *Dinodontosaurus* is one of the most common tetrapod taxon in the Triassic Santa Maria Supersequence. This genus has a complex taxonomic history and is represented in the Triassic of both Argentina and Brazil. Nevertheless, only the species *Dinodontosaurus tener* is currently recognized as being present in Brazil. *Dinodontosaurus tener* exhibits high morphological variability among known specimens, partly due to taphonomic alterations but also potentially reflecting intraspecific or even interspecific variation. This study evaluates the morphology and taxonomic assignment of specimen MCP‐1645‐PV, a relatively well‐preserved skull whose morphology was briefly described in the 1980s and at that time attributed to the genus *Chanaria*. Currently, *Chanaria platyceps* is considered synonymous with *Dinodontosaurus brevirostris*, a species regarded as endemic to Argentina. In this contribution, we reassess the morphology of specimen MCP‐1645‐PV and its possible classification as *Dinodontosaurus brevirostris*, in light of recent advances in the understanding of *Dinodontosaurus* taxonomy. Based on anatomical and morphometric comparisons, our study indicates the presence of *Dinodontosaurus brevirostris* in the Brazilian Triassic, highlighting the need for a critical reassessment of historical specimens.

## 1 INTRODUCTION

Dicynodonts (Anomodontia, Dicynodontia) were one of the dominant groups of terrestrial vertebrates during the Permian and Triassic periods (Angielczyk & Kammerer, 2018; Fröbisch, 2009; Kammerer & Ordoñez, 2021; King, 1993; Ray, 2006; Surkov & Benton, 2004). Belonging to the clade Therapsida, they were characterized mainly by a reduced (or even absent) dentition, generally a pair of tusks, an edentulous bony ‘beak’, and large temporal fenestrae—features that indicate herbivorous habits (Botha‐Brink & Angielczyk, 2010). Dicynodonts were among the tetrapods that survived the Permo‐Triassic mass extinction event, undergoing a radiation during most of the Triassic period (Botha & Smith, 2007; Vega‐Dias et al., 2004).

Dicynodonts had a worldwide distribution throughout their timespan (Fröbisch, 2009), with occurrences particularly common in the Karoo Basin, southern Africa (e.g., Smith et al., 2012). In South America, dicynodonts were particularly abundant from the Middle to Late Triassic, although they are also recorded in Permian rocks (Kammerer & Ordoñez, 2021). In Brazil, at least six genera of dicynodonts are considered as valid, and all of them are known exclusively from the southern part of the country, where tetrapod‐bearing Permian/Triassic rocks are concentrated. These include *Endothiodon* and *Rastodon*, both from Permian deposits of the Rio do Rasto Formation (Boos et al., 2013, 2016; Da Silva et al., 2023, 2025), and the Triassic genera *Dinodontosaurus*, *Stahleckeria*, *Sangusaurus*, and *Jachaleria* (Da Silva et al., 2023; Kammerer & Ordoñez, 2021; Schultz et al., 2020).

*Dinodontosaurus* are medium‐ to large‐sized kannemeyeriiform dicynodonts (Da Silva et al., 2023; Kammerer & Ordoñez, 2021) that were exceptionally abundant in the Middle to Late Triassic of Brazil. Despite their complex taxonomic history, only two species of this genus are currently considered as valid (Da Silva et al., 2023; Kammerer & Ordoñez, 2021): *Dinodontosaurus tener*, exclusive to Brazil, and *Dinodontosaurus brevirostris*, previously thought to be exclusive to Argentina. In Brazil, there is significant morphological variability in both cranial and postcranial samples of *Dinodontosaurus*, partially due to taphonomic alterations (e.g., compression or expansion due to diagenetic recrystallization; see Holz & Schultz, 1998) but also potentially reflecting intraspecific or even interspecific variations (see comments in Martinelli et al., 2017; Kammerer & Ordoñez, 2021; Escobar et al., 2021, 2023). For this reason, morphological assessments of these specimens are necessary.

In this context, this study examines the morphology and taxonomic assignment of specimen MCP‐1645‐PV, a relatively well‐preserved skull that was briefly described by Araújo (1981) and initially assigned to the genus *Chanaria*. In this contribution, we reassess the morphology of this specimen and its potential classification as *Dinodontosaurus brevirostris,* in light of current knowledge on the taxonomy of *Dinodontosaurus*.

Institutional abbreviations: *CRILAR‐Pv*, Centro Regional de Investigaciones Científicas y Transferencia Tecnológica, Paleontología de Vertebrados, Anillaco, La Rioja, Argentina; *MCP*, Museu de Ciências e Tecnologia of the Pontifícia Universidade Católica do Rio Grande do Sul, Porto Alegre, Rio Grande do Sul, Brazil; *MCT (=DGM)*, Museu de Ciências da Terra, Serviço Geológico do Brasil, Rio de Janeiro, Brazil; *MCZ VPRA*, Vertebrate Paleontology Collection, Museum of Comparative Zoology, Cambridge, United States; *MPDC (=MGB)*, Museu municipal Padre Daniel Cargnin, Mata, Rio Grande do Sul, Brazil; *PULR‐V (=UNLaR)*, Paleontología de Vertebrados, Museo de Ciencias Antropológicas y Naturales, Universidad Nacional de La Rioja, La Rioja, Argentina; *UFRGS*, Universidade Federal do Rio Grande do Sul, Porto Alegre, Rio Grande do Sul, Brazil.

## 2 MATERIALS AND METHODS

### 2.1 Specimen provenance

**FIGURE 1:**
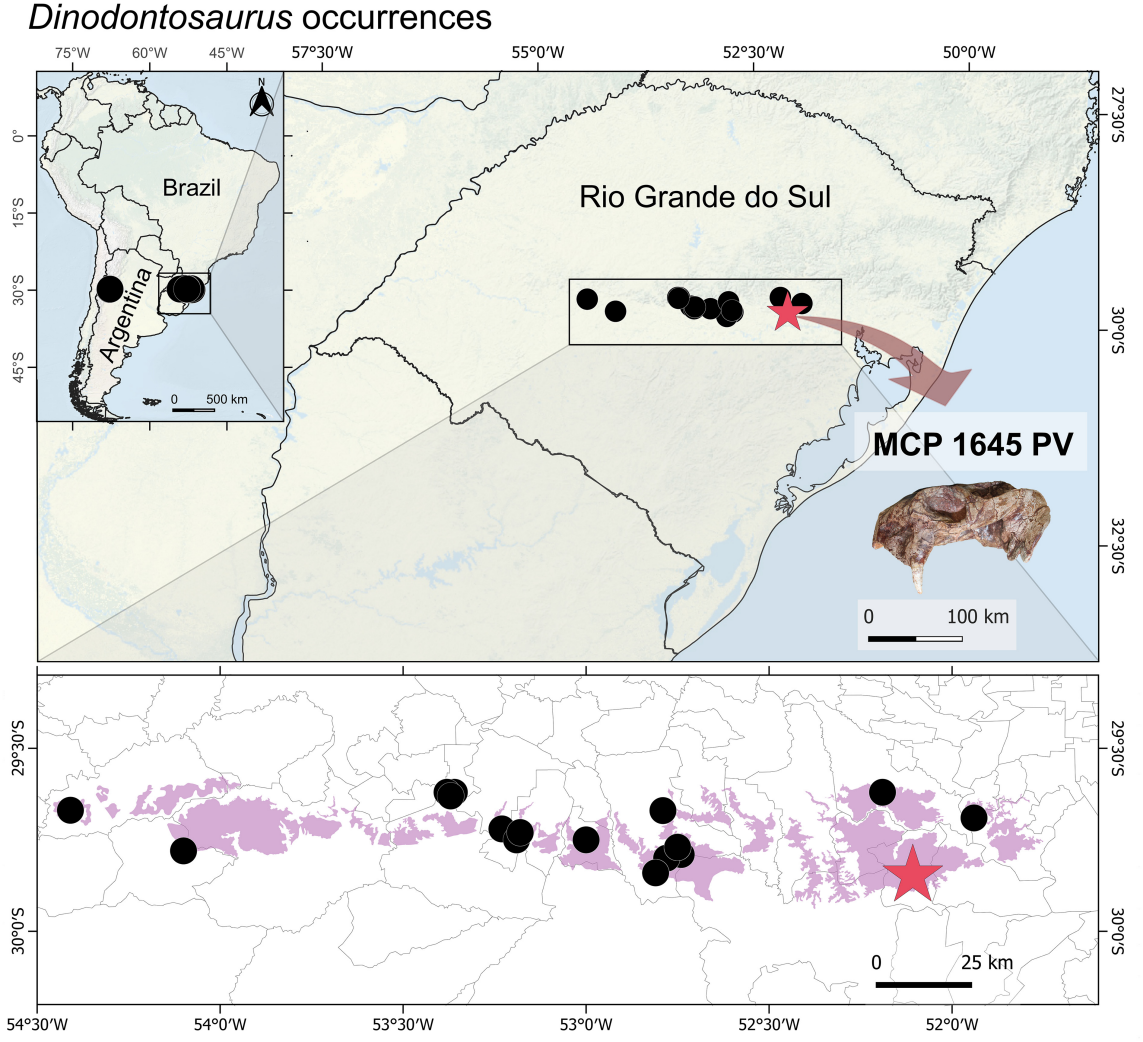
Map showing Brazilian and Argentine occurrences of specimens of the genus *Dinodontosaurus* (black dots), highlighting the collection site of MCP‐1645‐PV (red star). The purple shape represents the Triassic Santa Maria Supersequence.

Specimen MCP‐1645‐PV represents an almost complete and relatively well‐preserved skull. According to Araújo (1981) and collection records at MCP, it was collected in 1976 by the priestly brothers Daniel and Abrahão Cargnin. The specimen was recovered from a railway cut along the Porto Alegre–Santa Maria roadway, 3.5 km from the Professor Parreiras Station, southeast of the Melos district, in the municipality of Vale Verde, Rio Grande do Sul, Brazil (Figure 1). The specimen was prepared by Valdor Ochagávia da Costa and has since been housed in the paleontological collection of the MCP.

Although the Vale Verde region is relatively understudied, fossils collected from this area are characteristic of the *Dinodontosaurus* Assemblage Zone of the Santa Maria Supersequence. However, for a more in‐depth discussion on the variations within this biozone, see Martinelli et al. (2017). The Melos outcrops are stratigraphically equivalent to the Pinheiros‐Chiniquá Sequence of the Santa Maria Supersequence, which dates to the late Ladinian–early Carnian (Schultz et al., 2020). The main fossiliferous levels of the Pinheiros‐Chiniquá sequence correspond to thick layers of massive or laminated red mudstones, lying above planar/cross‐stratified conglomeratic sandstones. This succession indicates the progression from a high‐energy fluvial canal into lake deposits (Horn et al., 2013). Although it is probable that MCP‐1645‐PV comes from the massive siltstone layers of the Pinheiros‐Chiniquá sequence, the specimen lacks accurate field information.

### 2.2 Photogrammetry

Specimen MCP‐1645‐PV was photographed using a Canon T7 Rebel camera to compose a three‐dimensional model through photogrammetry. The image acquisition protocol involved 360° photography at three distinct levels, ensuring an approximately 80% overlap between images to achieve complete and detailed coverage of morphological structures.

To maintain morphological accuracy, appropriate ambient lighting was used to prevent visual distortions, and a metric scale was included to accurately record the fossil's dimensions. After image capture, the photographs were imported into Agisoft Metashape and a dense point cloud was made, based on which a 3D mesh was constructed and refined to enhance detail.

This methodology enables precise scientific analysis and contributes to specimen preservation by minimizing the need for physical handling, thereby reducing the risk of damage or breakage. Following 3D modeling, a detailed anatomical description of the relevant cranial elements was conducted.

### 2.3 Data acquisition and statistical analysis

**FIGURE 2:**
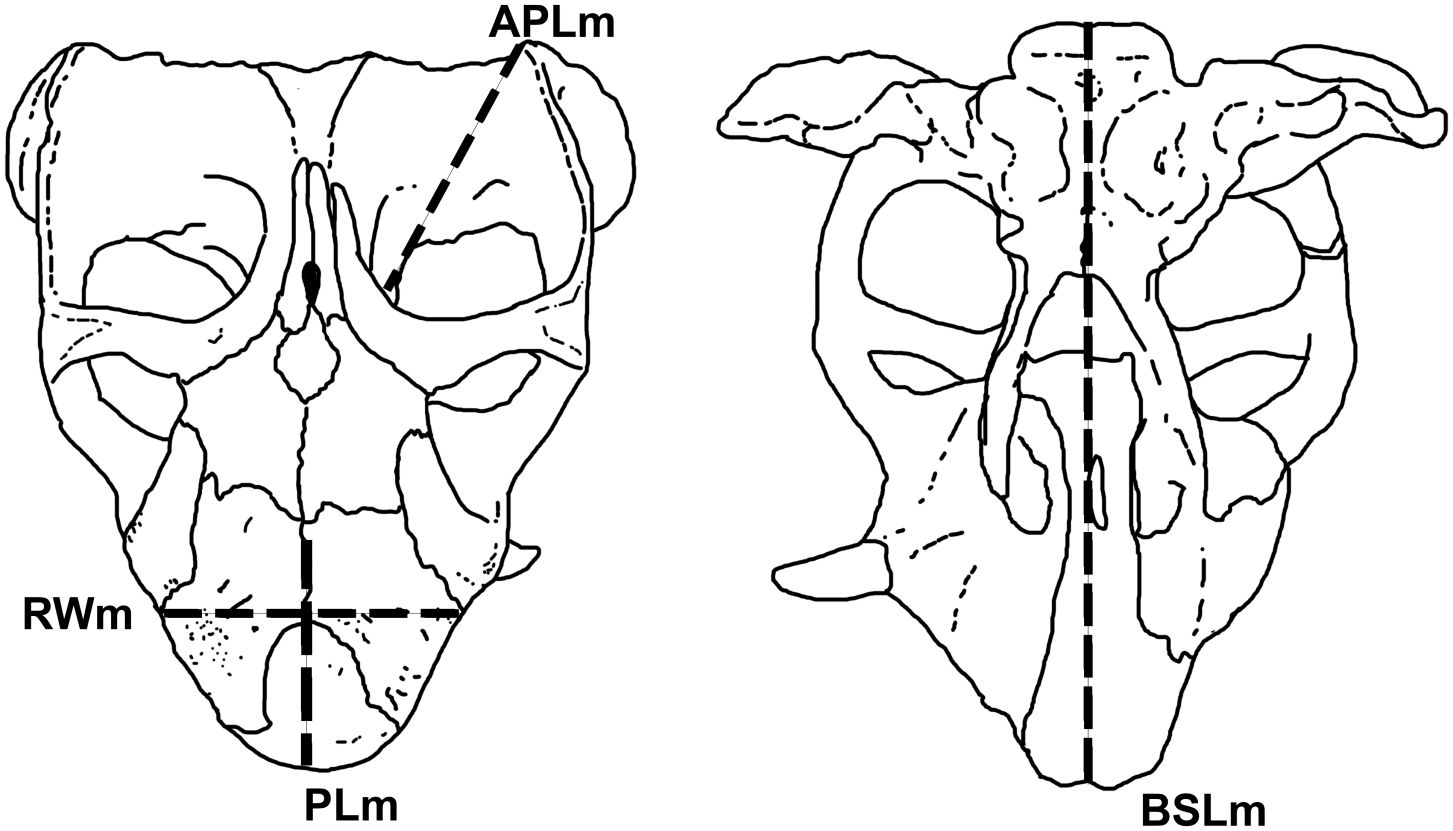
Measurements taken in MCP‐1645‐PV in dorsal view (left) and ventral view (right): rostral width measurement (RWm), pre‐orbital length (PLm), anteromedial‐posterolateral length of the temporal fenestrae (APLm), and basal skull length measurement (BSLm).

We took pictures of 15 specimens (Table 1)—comprising both *Dinodontosaurus tener* and *Dinodontosaurus brevirostris*—and utilized the software ImageJ to obtain precise linear measurements (Figure 2), including: (i) basal skull length; (ii) pre‐orbital length (measured from a point level with the anterior margin of the orbit); (iii) rostral width (measured at the midpoint of the rostrum's length); and (iv) anteromedial‐posterolateral length of the temporal fenestrae. Measurements (ii)–(iv) were standardized against basal skull length to ensure proper comparability. Additionally, we incorporated specimen continuous data provided by Kammerer and Ordoñez (2021). For a full list of specimens and measurements, please refer to Data S2, Supporting Information.

**TABLE 1:** Specimens used in the anatomical and morphometric comparison.

*Dinodontosaurus tener*	*Dinodontosaurus brevirostris*
UFRGS‐PV‐0228‐T	PULR‐V 14
UFRGS‐PV‐0227‐T	PULR‐V 15
MCP‐128c‐PV	MCZ VPRA‐3453
MCP‐266a‐PV	MCP‐1645‐PV
MCP‐648‐PV	CRILAR‐Pv 94
MCP‐531c‐PV	PULR‐V 03
MCP‐737‐PV	
MPDC 592‐126	
UFRGS‐ PV‐0621‐T	

To investigate the modality of cranial ratios measured, we applied two complementary statistical approaches: Hartigan's dip test for unimodality and Bimodality Coefficient (BC). Hartigan's dip test is a non‐parametric test designed to detect departures from unimodality in a univariate distribution. The test evaluates the maximum difference between the empirical cumulative distribution function of the observed data and the best‐fitting unimodal distribution. A *p*‐value less than 0.05 is typically interpreted as evidence against unimodality, suggesting that the data may follow a bimodal or multimodal distribution. The test was implemented in R using the function *dip. test()* from the “diptest” package (Maechler, 2016).

To complement the hypothesis‐based test, we also computed the Bimodality Coefficient, which combines information from the third and fourth standardized moments of the distribution, skewness and excess kurtosis, respectively. The BC was calculated with function *bimodality_coefficient()* in package “mousetrap” (Kieslich & Henninger, 2017); this function calculates the BC based on the procedures described in Pfister et al. (2013), which include corrections for sample biases in skewness and excess kurtosis. A BC value greater than 5/9 (≈0.555) is typically interpreted as indicative of bimodality, whereas lower values are consistent with unimodal distributions.

Together, these two approaches—one inferential and one descriptive—provide a robust framework for identifying potential bimodality in the distribution of continuous morphological variables and, by extension, for inferring any potential morphological differentiation among the taxa under study. To assess statistical differences between species means, we applied Student's *t* test, with the null hypothesis stating that the difference between group means is equal to zero. Plots were generated using the “ggplot2” package (Wickham, 2016). All statistical analyses were performed in the R programming environment (R Core Team, 2024).

### 2.4 Phylogenetic analysis

To investigate the phylogenetic position of specimen MCP‐1645‐PV, we scored it in the phylogenetic data matrix published by Mueller et al., 2023, which is an updated version of the Kammerer and Ordoñez (2021) matrix. We also included new scorings of *Rastodon procurvidens* based on Da Silva et al. (2025). We followed the strategy proposed by Mueller et al. (2023) for the maximum parsimony analyses. First, we performed an analysis with all 121 taxa and 199 characters (23 continuous +176 discrete); *Biarmosuchus tener* was set as the outgroup. Second, we performed a new analysis on a reduced data matrix, consisting mostly of well‐sampled kannemeyeriiform taxa. This approach excluded most non‐kannemeyeriiform dicynodonts and, characters not informative for resolving relationships within Kannemeyeriiformes. Nevertheless, *Aulacephalodon bainii* (set as outgroup), *Dicynodon angielczyki*, *Lystrosaurus declivis*, and *Lystrosaurus murrayi* were included as outgroup taxa for comparative purposes. All parsimony‐based phylogenetic analyses were run in TNT v.1.5 (Goloboff & Catalano, 2016). The parameters are described below.

As an initial step, we allowed the inclusion of suboptimal trees within 10 steps from the most parsimonious score, with a relative fit threshold of 0.1. This approach enables broader exploration of the tree space and avoids premature convergence on local optima. Subsequently, we performed a traditional search with the TRB algorithm, using 1000 random addition sequences, saving up to 100 trees per replicate. This step aims to ensure a comprehensive sampling of the most parsimonious topologies. After this phase, suboptimal trees were discarded, and a final round of tree searching was carried out using the trees retained in RAM. This additional step refines the search within the space of optimal or near‐optimal trees, maximizing the likelihood of recovering the most accurate phylogenetic hypotheses. The most parsimonious tree obtained was then plotted with the help of Chiplot (Xie et al., 2023). When calculating the consensus trees, nodes with minimum branch length = 0 were collapsed. Absolute Bremer indexes were calculated based on trees suboptimal by 10 steps retained during the first phase of the search.

We also conducted a Bayesian phylogenetic analysis using *MrBayes* v3.2.7a (Ronquist et al., 2012) to investigate the evolutionary relationships among the included taxa. The outgroup was designated as *Biarmosuchus tener* as in the parsimony analysis for all taxa; for the reduced dataset, *Aulacephalodon bainii* was set as the outgroup. We employed the Markov k‐state model with a gamma‐distributed rate variation across characters (Lewis, 2001). To accommodate heterogeneity in character change rates, we enabled variable rates prior. Four independent MCMC runs were executed in parallel, each comprising four chains (one cold, three heated) and two swap attempts per cycle. The analysis was run for 30 million generations, sampling every 1000 generations. We used a relative burn‐in of 50%, thus discarding the first half of samples. Convergence diagnostics were monitored through the average standard deviation of split frequencies (~0.01) and potential scale reduction factors (PSRF close to 1). To determine whether the runs reached the stationary phase and to ensure that the effective sample size (ESS) for each parameter was greater than 200, we used the *Tracer v. 1.6* software (Rambaut et al., 2014). Both the extended majority and majority‐rule consensus trees were produced and saved separately.

## 3 RESULTS

### 3.1 Systematic paleontology

Therapsida (Broom, 1905).

Dicynodontia (Owen, 1859).

Bidentalia (Owen, 1876).

Kannemeyeriiformes (Maisch, 2001).

### 3.2 *Dinodontosaurus* (Romer, 1943)

Type species: *Dinodontosaurus oliveirai* Romer (1943) (=*Dicynodon tener*; von Huene, 1935).

Diagnosis: According to Kammerer and Ordoñez (2021), a medium to large (~40 cm maximum skull length) kannemeyeriiform dicynodont characterized by the combination of a tab‐like median anterior process of the frontals, a postorbital bone bearing an elongate, curved suborbital process, and an elongate caniniform process sharply offset from the zygomatic arch. A hypertrophied tusk is present in large individuals.

### 3.3 *Dinodontosaurus brevirostris* (Cox, 1968)

**FIGURE 3:**
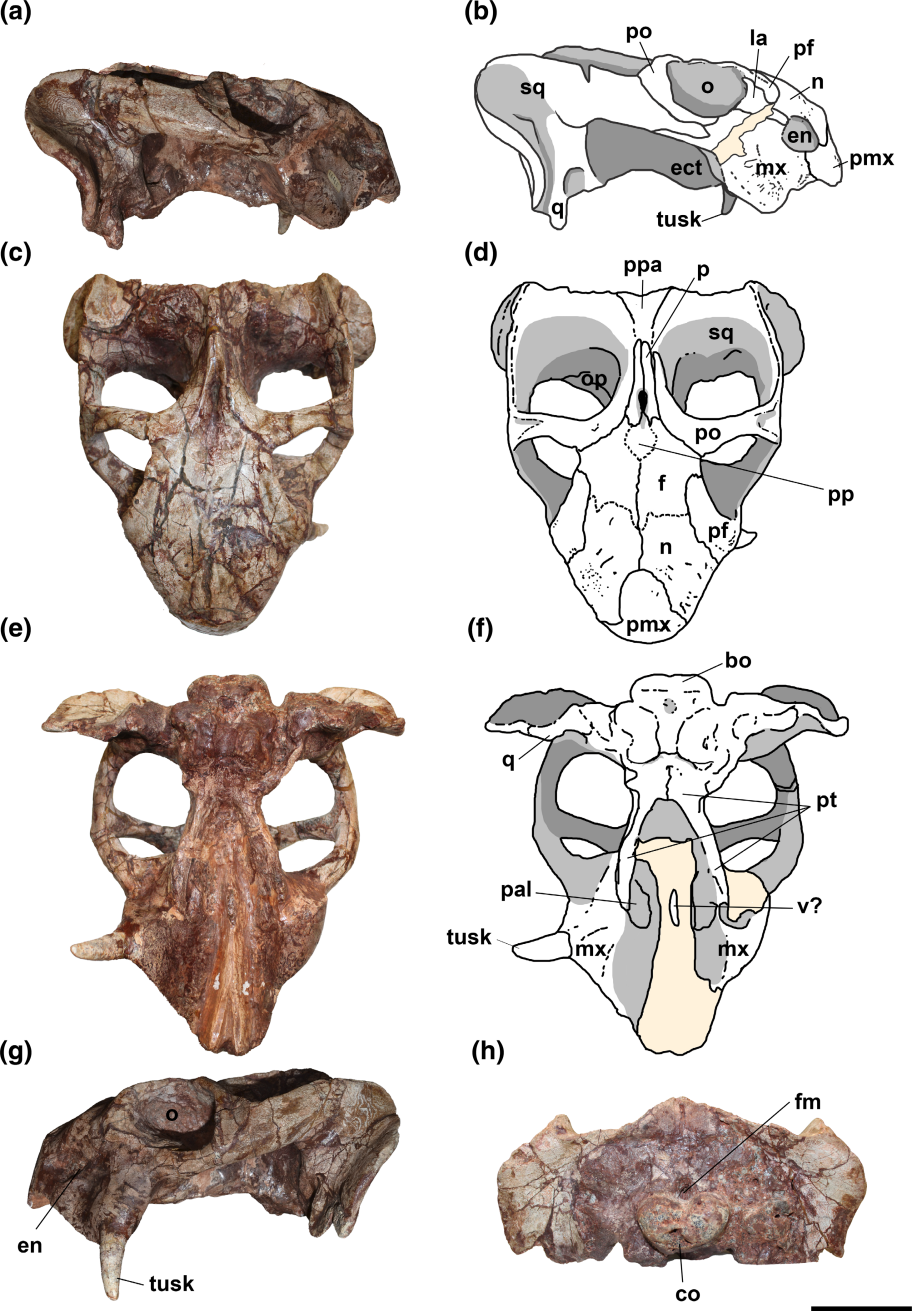
Skull of the Brazilian *Dinodontosaurus brevirostris* specimen MCP‐1645‐PV in right lateral (a), dorsal (c), ventral (e), left lateral (g) and occipital (h) views. Interpretative drawings of the right lateral (b), dorsal (d) and ventral (f) views. Scale: 5 cm. bo, basioccipital; co, occipital condyle; ect, ectopterygoid; en, external nares; f, frontal; fm, foramen magnum; la, lacrimal; mx, maxilla; n, nasal; o, orbit; op, opisthotic; p, parietal; pal, palatine; pf, prefrontal; pmx, premaxilla; po, postorbital; pp, preparietal; ppa, postparietal; pt, pterygoid; sq, squamosal; q, quadrate; v?, possible vomer. Portions in beige represent regions restored with plaster.

Referred specimen: MCP‐1645‐PV, a nearly complete, well‐preserved skull (Figure 3).

Diagnosis: According to Kammerer and Ordoñez (2021), *Dinodontosaurus brevirostris* is distinguished from *Dinodontosaurus tener* by having a proportionally broader skull, with a triangular intertemporal bar. This bar is expanded anteriorly around the pineal foramen, gradually narrowing posteriorly, forming only a narrow crest at its posterior end.

### 3.4 Description

The skull is nearly complete, but some regions have been reconstructed with plaster (Figure 3). This includes most of the ventral surface of the premaxillaeo (Figure 3e,f), including the anterior palatal ridges and the posterior median ridge. Parts of the right maxilla and lacrimal also exhibit plaster restorations. The occipital region is severely damaged, and, except for the basioccipital and the lateral portions of the squamosal, little information can be extracted from it. The specimen also presents numerous cracks throughout the skull, which are filled with sediment or adhesive.

#### 3.4.1 Premaxillae

The premaxillae are fused, forming a single continuous structure in the anterior region of the skull, as is common in dicynodonts. This element exhibits evidence of polishing marks resulting from overpreparation, which have impacted the preservation of some morphological details. For instance, in palatal view, the anterior ventral ridges of the premaxillae have been entirely reconstructed. However, the notch observed in anterior view attests to the original presence of these structures. The premaxilla forms the anteroventral borders of the external nares (Figure 3a,b). Laterally, the premaxilla contacts the maxilla (Figure 3a,b), with the suture between these elements displaying an interdigitated pattern and a dorsoventral orientation in lateral view. Additionally, there is a posterodorsal contact with the nasals, where the ascending process of the premaxilla is positioned between the anterior portions of those bones. Preservation does not allow for a clear identification of the septomaxillae. The suture lines between the premaxilla and the nasals, in dorsal view (Figure 3c,d), are oriented from anterolateral to posteromedial. The ascending process of the premaxilla, seen dorsally, has a triangular shape. In anterior view, the ventral limit of the premaxilla exhibits a “W”‐shaped configuration, forming the previously mentioned notch. The bone shows visible ornamentation in the shape of small foramina or grooves, which are concentrated mainly in its anteroventral portion but are also distributed—though less densely—across the rest of the bone surface. However, the polishing process during previous preparation has hindered the detailed observation of this ornamentation in some regions.

#### 3.4.2 Maxillae

The maxillae also exhibit polishing marks associated with overpreparation, and on the right side, there is evidence of some reconstruction, especially in its lateral and ventral surfaces (Figure 3a,b,e,f). Despite these interventions, the left side provides a clearer view of the original morphology of the maxilla. The maxilla is one of the most conspicuous bones of the skull and occupies a prominent position in facial anatomy, being the dominant element in lateral view (Figure 3a,b,g). Anteriorly, the maxillae articulate with the premaxilla, while dorsally, they contact the lacrimals (Figure 3a,b). The zygomatic process of the maxilla bifurcates to accommodate the anterior portion of the zygomatic process of the squamosal. The maxillae possess well‐developed caniniform processes, which are triangular in lateral view and house the tusks. In lateral view, these processes protrude ventrally from the maxilla and they are markedly offset from the ventral margin of the zygomatic process of the maxilla. This condition is a diagnostic feature of the genus *Dinodontosaurus* (Kammerer & Ordoñez, 2021). The anterior margins of the caniniform processes form an acute angle of 40.1° with the longitudinal axis of the skull. In its posterior portion (as measured from the left side of the skull), the initial angle is 97.8°, and more distally, it changes to 119.1°.

The maxillae are covered in foramina, mainly on the surface of the caniniform process. The surface of the premaxilla between the external nares and the lateral buttress of the caniniform process is depressed, forming a “groove” that runs anteromedially to dorsoposteriorly. Between the external nares and the embayment that houses the caniniform process, the maxillary surface is grooved. This groove begins approximately at the midpoint of the anteroventral border of the maxilla and extends posterodorsally, reaching approximately the level of the posterior border of the external nares. The maxillary dentition is reduced to tusks only, although only the left one is preserved in the specimen. It is a moderately developed tusk, oriented vertically relative to the skull axis. The eruption point of the tusk is approximately level with the anterior margin of the orbit.

#### 3.4.3 Nasals

Although both nasals are preserved, the right counterpart is in better condition, showing fewer signs of structural damage or reconstruction. Based on our interpretation, the lacrimal prevents the nasal from establishing ventral contact with the maxilla (Figure 3a,b,g). In *Dinodontosaurus*, this contact has been variably reported as present or sometimes absent (see Cox, 1965, 1968; Morato, 2006). Anteriorly, the nasals contact the premaxillae and, dorsolaterally, they articulate with the prefrontals (Figure 3c,d). The sutures between the nasals and the prefrontals have a laterally arched orientation, while anteriorly, the nasals are arranged in a way that forms an inverted “V” medially, accommodating the ascending process of the premaxilla. Posteriorly, they articulate with the frontals, and ventrally, they contact the lacrimal near the junction with the prefrontal (Figure 3a,d). The nasals contribute to forming the dorsal border of the external nares. The nasal ornamentation is notable, primarily consisting of foramina, which are concentrated in the dorsal region of the external nasal opening.

#### 3.4.4 Lacrimals

The lacrimals form the anterior borders of the orbits (Figure 3a,b). In lateral view, each lacrimal contacts the maxilla ventrally, the prefrontal dorsally, and the jugal posteriorly, with the contact between the lacrimal and jugal being primarily observed on the anterior internal surface of the orbit. The lacrimals extend anteriorly, presumably meeting the septomaxillae at the posterior margins of the external nares, as reported in several specimens of *Dinodontosaurus* (Cox, 1968; Morato, 2006). The suture line between the lacrimals and maxillae is predominantly anteroposteriorly oriented.

#### 3.4.5 Prefrontals

The left side of the skull preserves the prefrontal bone more completely, allowing for a clearer observation of its articulations and morphology. In the dorsal view, the posterior margin of the prefrontal forms the anterior and anteromedial borders of the orbit. The prefrontal has a crescent shape, characteristic of its contribution to the curvature of the anterior orbital rim. Medially, it contacts the frontal bone, while anteromedially, it articulates with the nasal. In lateral view, the prefrontal contacts the lacrimal posteroventrally. The bone exhibits some cracks and polishing marks.

#### 3.4.6 Frontals

The frontals are located in the cranial roof connecting anteriorly with the nasals, anterolaterally with the prefrontals, and posterolaterally with the postorbitals (Figure 3a,b). Posteriorly, there is a presumed contact with the parietals and an observable contact with the preparietal. The sutures between these bones are difficult to discern, but the suture with the frontal is arched anterolaterally where it meets the posterior portion of the prefrontal, while the suture with the postorbital is slightly arched posteriorly, following an anterolateral‐to‐posteromedial orientation. In several specimens of *Dinodontosaurus*, the frontals exhibit a “tab‐like” anterior medial process, a diagnostic feature of the genus according to Kammerer and Ordoñez (2021). Although in MCP‐1645‐PV this area is not well preserved, an anterior process is conspicuous in the midline of the frontonasal suture. However, it has an oval or “tongue”‐like shape, rather than the “tabular” shape described by Kammerer and Ordoñez (2021). The frontal bone lacks ornamentation but contributes to forming the dorsal margin of the orbit.

#### 3.4.7 Postorbitals

The postorbitals (Figure 3a–d) bear two processes: the descending process, which forms the postorbital bar, and the posterior process, which extends along the intertemporal bar, forming the anteromedial border of the temporal fenestra. In lateral view, at the suborbital bar, the descending process of the postorbital contacts the jugal medially and the squamosal ventrally (Figure 3a,b). In the dorsal view, the postorbital contacts the frontal anteromedially, and its posterior process contacts the parietal posteromedially along nearly half the length of the intertemporal bar (Figure 3c,d). The suture with the parietal is primarily directed anteroposteriorly, with a slight medial component towards the end of the posterior process of the postorbital. The suture with the frontal is slightly arched posteriorly, extending posteromedially from the posteromedial border of the orbit. The suture with the squamosal is ventrally convex, determining an elongated and curved suborbital process of the postorbital—a diagnostic feature of *Dinodontosaurus* (Kammerer & Ordoñez, 2021). There is no evidence of ornamentation on the lateral surface of the postorbital bar, although this could be related to the poor preservation and overpreparation in this region.

#### 3.4.8 Preparietal

The preparietal is an unpaired bone located on the cranial roof and contacts the frontals anteriorly and the parietals, although the sutures are not clearly visible in the specimen (Figure 3c,d). The overall shape of the bone is not clearly discernible, but the preparietal region is depressed relative to the surrounding bones, suggesting a slight recess in its position. The preparietal contributes to the boundaries of the pineal foramen, forming its anterior margin. The pineal foramen is oval, being longer anteroposteriorly than it is wide, with its lateral and posterior borders formed by the parietals. It is positioned posterior to the base of the postorbital. The preparietal exhibits some cracks on its surface, likely due to preservation.

#### 3.4.9 Parietals

The posterior region of the parietals exhibits some fractures, but overall, both bones are well preserved (Figure 3c,d). The parietals are the primary components of the intertemporal region of the skull. They articulate posteriorly with the postparietal, anteriorly with the preparietal, and laterally with the posterior processes of the postorbitals. At the posteromedial portions of the temporal fenestrae, the parietal contacts with the squamosals in a more ventral position, as dorsally the postparietal prevents direct contact between these bones. Both parietals form a “V”‐shaped suture with the postparietal, while the sutures with the postorbitals are mainly oriented anteroposteriorly. The overall shape of the parietals in dorsal view is that of an elongated, roughly isosceles triangle (wider anteriorly, narrower posteriorly). The angle formed between the lateral margins of the parietals and the sagittal plane is of 10.7°. The anterior part of the intertemporal bar surrounding the pineal foramen is more expanded, while the posterior part narrows towards its contact with the postparietal, giving the intertemporal bar a triangular aspect, a diagnostic feature of *Dinodontosaurus brevirostris* (Kammerer & Ordoñez, 2021). The parietals form the posterior and lateral margins of the pineal foramen.

#### 3.4.10 Postparietal

The postparietal is located at the terminal portion of the intertemporal region of the skull (Figure 3c,d). Its overall shape is approximately triangular, with curved lateral edges. It articulates anterolaterally with the parietals and posterolaterally with the squamosals. Presumably, the postparietal also contacts the supraoccipital ventrally, but this is not conspicuous in MCP‐1645‐PV (see Cox, 1965, 1968; Morato, 2006). In the dorsal view, the anterior end of the postparietal is wedged between the terminal portions of the parietals.

#### 3.4.11 Squamosals

The squamosals (Figure 3a–d) are characterized by three distinct processes: the quadrate process, which extends dorsoventrally; the zygomatic process, oriented anteroposteriorly; and the temporal process, directed lateromedially. The zygomatic and temporal processes form the lateral and posterior surfaces of the temporal fenestrae, respectively. Ventral to the postorbital bar, the zygomatic process of the squamosal contacts the suborbital process of the postorbital. Below the orbit, the zygomatic process projects anteriorly in a triangular shape and is received by the bifurcated posterior process of the maxilla. In the intertemporal region, the temporal process of the squamosal contacts the parietal and postparietal medially. In lateral view, the squamosal is positioned lateral to the jugal, though fractures make it difficult to identify the suture. The quadrate process of each squamosal extends ventrally, exhibiting a sinuous shape in the left lateral view. The pronounced curvature of the lateral flange of the quadrate process, together with the lateral surface of the zygomatic process, creates a deep concavity, interpreted as a support area for the *M. adductor mandibulae externus lateralis* (Angielczyk et al., 2018; Crompton & Hotton III, 1967; Ordoñez et al., 2020). The sutures with the postorbitals have a diagonal orientation, running from anteroventral to posterodorsal in lateral view. The jugal lies medially to the zygomatic process of the squamosal.

#### 3.4.12 Pterygoids

Although partially reconstructed, the pterygoids exhibit the typical X‐shape (Figure 3e,f), with their palatal processes—which are longer than the quadrate processes—projecting anterolaterally, while the quadrate processes extend posterolaterally at a wider angle.

#### 3.4.13 Palatine

Medial to the anterior margin of the left palatal process of the pterygoid, the left palatine is preserved, being longer than wide.

#### 3.4.14 Occiput bones

The occipital surface is severely damaged, preventing an assessment of the morphology of the opisthotics and the supraoccipital. Among the few elements that can be identified is the basioccipital (Figure 3h), articulating dorsally with the exoccipitals (in the occipital condyle) and anteriorly with the basisphenoid (in the basicranium). In ventral view, the basisphenoid‐basioccipital suture forms a posteriorly arched shape just anterior to the basal tubera. A foramen is present on the left side of the basisphenoid, which may be one of the foramina for the carotid artery. The basioccipital exhibits robust basal tubera, which are longer than they are wide. The stapedial facets are visible, particularly on the right side, and are oriented ventrolaterally. The region between the basal tubera is smooth, lacking an intertuberal crest, which is observed in other *Dinodontosaurus* specimens, such as an Argentine specimen described by Ordoñez et al. (2020). The shape of the occipital condyle is roughly kidney‐ or bean‐like in posterior view.

#### 3.4.15 Dentition

Only the left tusk is preserved, with its crown erupted (Figure 3g). In anterior view, the tusk projects ventrolaterally, while in lateral view it is subvertically oriented. It is moderately developed, slightly longer than the caniniform process itself. The tusk is conical, tapering ventrally until it terminates in a rounded tip.

### 3.5 Cranial proportions

**FIGURE 4:**
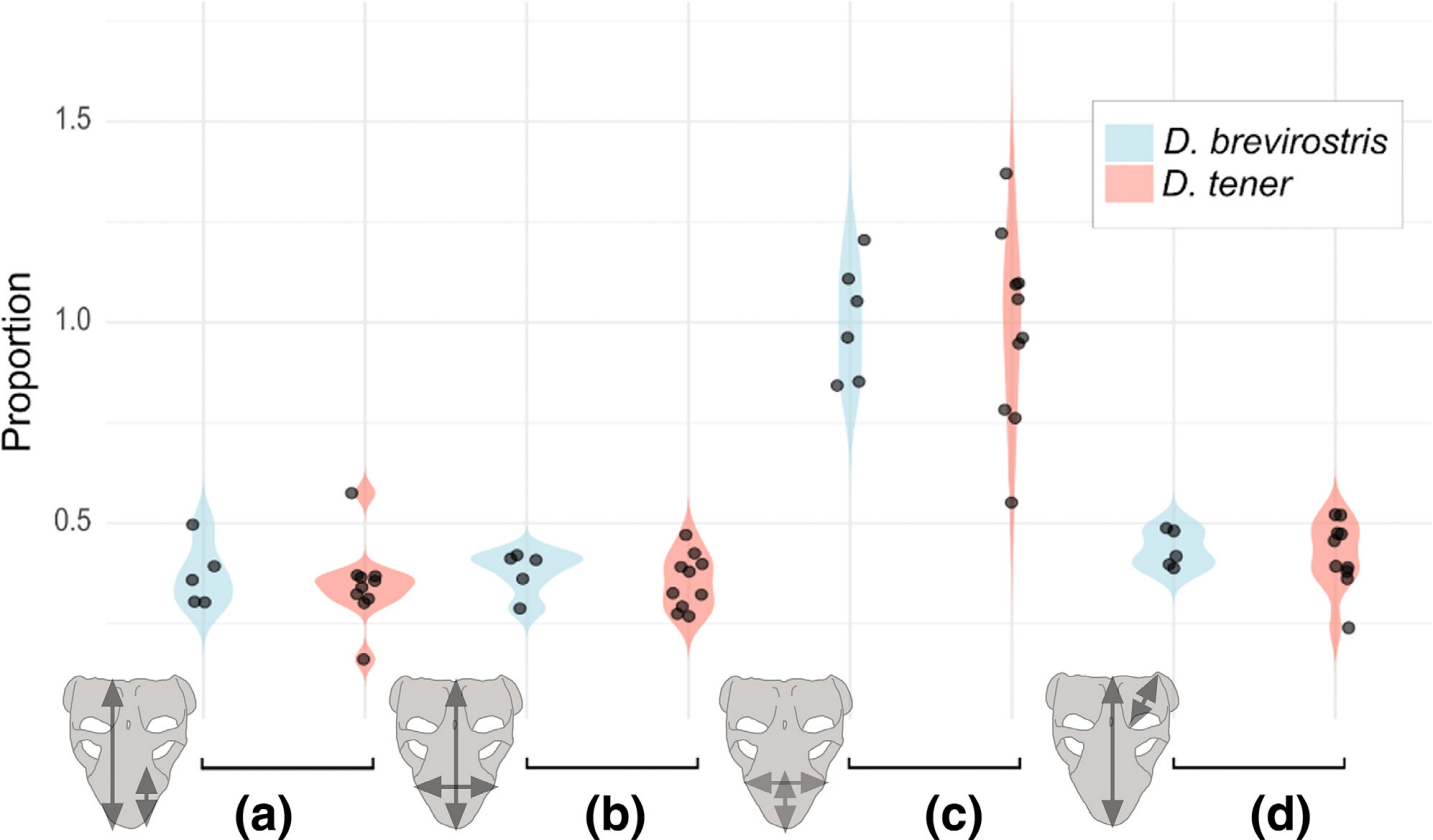
Violin plots comparing anatomical proportions of *Dinodontosaurus brevirostris* and *Dinodontosaurus tener*. The shaded areas represent the distribution of values for each species: blue for *Dinodontosaurus brevirostris* and salmon for *Dinodontosaurus tener*. Panels show: (a) relative length of the preorbital region with basal length; (b) relative width of the rostrum with basal length; (c) ratio of preorbital length to rostrum width; (d) relative length of the fenestra with basal length.

The distribution of the proportions studied is summarized in Figure 4. The mean pre‐orbital region length relative to the basal skull length is 6% greater in *Dinodontosaurus brevirostris* compared to *Dinodontosaurus tener*. However, this difference is minimal and not statistically significant (*p*‐value = 0.6307). It is also worth noting the considerable variation in this proportion among the sampled specimens of *Dinodontosaurus tener*. The mean relative width of the rostrum in *Dinodontosaurus brevirostris* is approximately 6% greater than in *Dinodontosaurus tener*, although this difference is also not statistically significant (*p*‐value = 0.4966). The mean ratio between the length of the preorbital region and the width of the rostrum in *Dinodontosaurus tener* is only 2% lower than in *Dinodontosaurus brevirostris*, and once again, this difference is not statistically significant (*p*‐value = 0.8435). Finally, the mean relative length of the fenestra (measured in the anteromedial‐to‐posterolateral direction) in *Dinodontosaurus brevirostris* is 3.2% greater than in *Dinodontosaurus tener*, although the null hypothesis cannot be rejected (*p*‐value = 0.7004). Additionally, the median value of this proportion is higher in *Dinodontosaurus tener* than in *Dinodontosaurus brevirostris*, and both the maximum and minimum values are more extreme for *Dinodontosaurus tener* compared to *Dinodontosaurus brevirostris* (Figure 4d).

**FIGURE 5:**
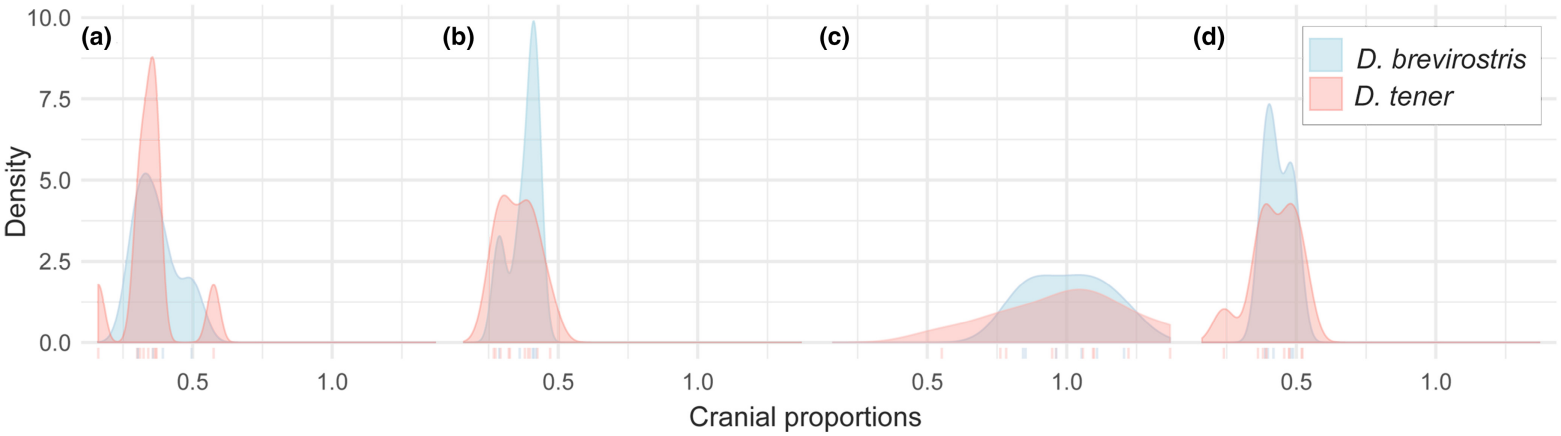
Density curves comparing the cranial anatomical proportions of *Dinodontosaurus tener* and *Dinodontosaurus brevirostris*. The shaded areas represent the distribution of each species: blue for *Dinodontosaurus brevirostris* and red for *Dinodontosaurus tener*. (a) Relative length of the preorbital region; (b) relative width of the rostrum; (c) preorbital length/rostrum width ratio; (d) relative length of the fenestra. The differences in distributions reflect interspecific variation, with some overlap in certain metrics and clear distinctions in others.

Figure 5 summarizes the overlap of measurements for each species. Regarding the relative length of the preorbital region (Figure 5a), *Dinodontosaurus tener* exhibits a higher density at lower values of this metric, while *Dinodontosaurus brevirostris* dominates the intermediate and higher values. This pattern suggests differences in cranial proportions between the two species, possibly reflecting distinct functional adaptations. In terms of the relative width of the rostrum (Figure 5b), *Dinodontosaurus brevirostris* shows a concentration of higher values with a pronounced density peak, whereas *Dinodontosaurus tener* presents a broader distribution with greater density at median values. This difference indicates that *Dinodontosaurus brevirostris* has a proportionally wider rostrum.

As has been shown, there is broad overlap between species proportions; hence, we should not expect that a joint distribution for the examined proportions would reveal a bimodal distribution. Indeed, Hartigan's dip test and bimodality coefficients show no support for a bimodal distribution (Table 2).

**TABLE 2:** Summary of Hartingan's dip test and bimodality coefficient calculated.

Proportion	Dip test (*p*‐value)	Bimodality coefficient
p1	0.2636736	0.2128380
p2	0.5182679	0.3949292
p3	0.5095853	0.2704169
p4	0.1848152	0.3545371

*Note*: Proportions are as follows: p1, pre‐orbital length/basal skull length; p2, mean rostrum width/basal skull length; p3, pre‐orbital length/mean rostrum width; p4, average fenestra length/basal skull length. For the dip tests, *p*‐values over 0.05 are interpreted as support against bimodality. A BC value greater than 5/9 (≈0.555) is typically interpreted as indicative of bimodality.

### 3.6 Phylogenetic results

**FIGURE 6:**
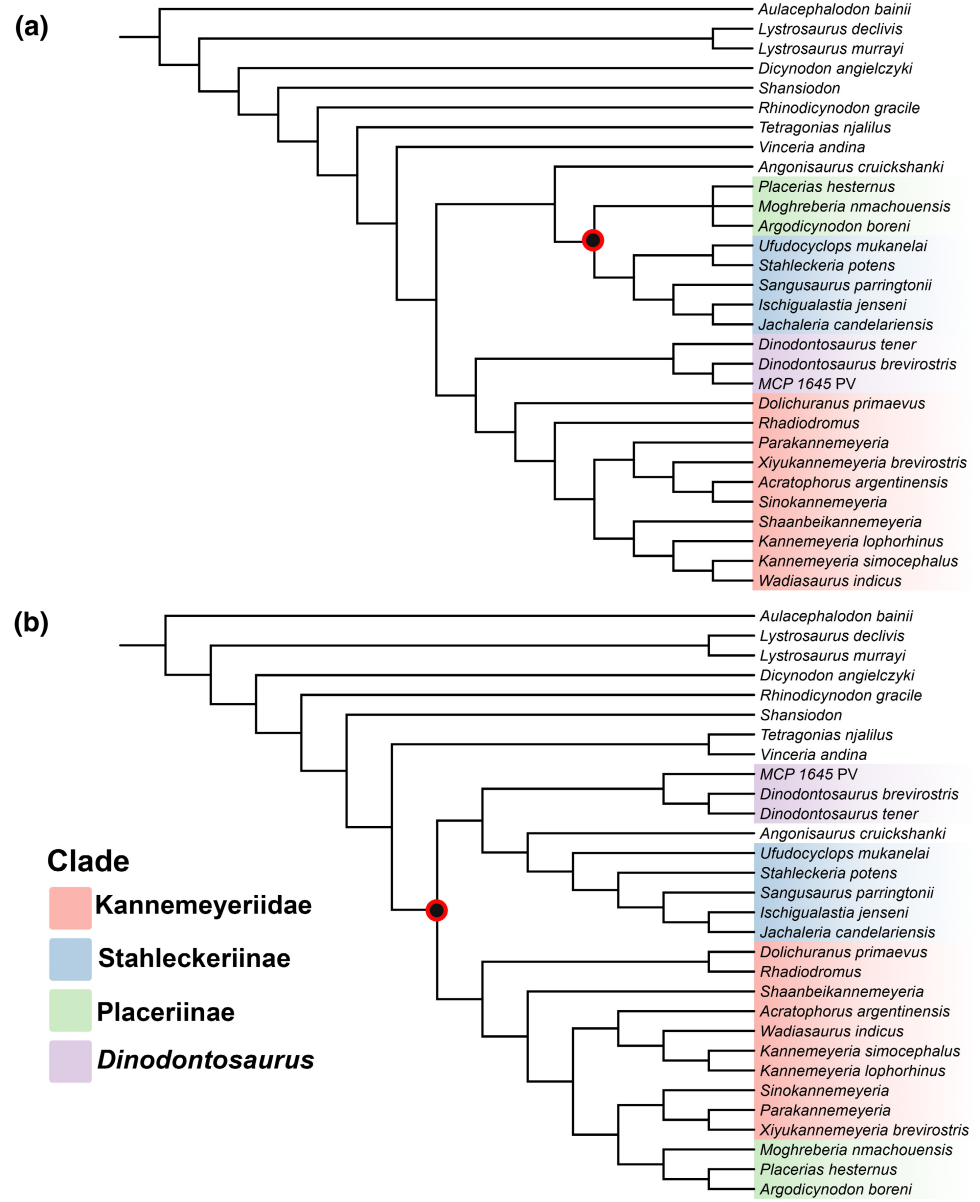
Results of the phylogenetic analyses. MCP 1645 PV groups with *Dinodontosaurus* in all analyses performed. (a) Strict consensus tree found under parsimony analysis (reduced dataset) (including continuous scorings of MCP 1645 PV); (b) extended majority consensus under Bayesian analysis (reduced dataset). The black and red dot marks the Stahleckeriidae node. Branch lengths and node support values were omitted for visualization purposes.

Bayesian and Parsimony analyses recovered MCP‐1645‐PV in a position consistent with its attribution to the genus *Dinodontosaurus*. Figure 6 shows only the results of the analyses performed on the reduced dataset that we used to infer phylogenetic relationships among kannemeyeriiform dicynodonts. In the strict consensus tree resulting from the two maximum parsimony trees (length = 328.889) found by the search, the *Dinodontosaurus* node is supported by the following synapomorphies (reduced dataset): presence of tusks (Char. 45: 1 → 2); development of the anterior median process of the frontals (Char. 58: 0 → 1); frontals develop a broad contribution to the dorsal rim of the orbits (Char. 63: 1 → 0). Parsimony analysis (reduced dataset, Figure 6a) recovered MCP‐1645‐PV as sister to *Dinodontosaurus brevirostris*. Only continuous characters support this: change in the relative length of the preorbital region of the skull (Char. 1: 0.339–0.356 → 0.321); relative changes in the width of the pterygoid median plate (Char. 8: 0.143 → 0.162); and change in the angle formed by the posterior pterygoid rami (Char. 9: 7.300–7.500 → 9.000). Absolute Bremer support for the MCP‐1645‐PV + *Dinodontosaurus* brevirostris is low (0.300).

The Bayesian inference based on the reduced dataset, which lacks continuous characters in this case due to software limitations, unsurprisingly recovered *Dinodontosaurus* brevirostris and *Dinodontosaurus tener* as sister taxa (posterior probability = 0.79), with MCP‐1645‐PV recovered as sister to them on the extended majority consensus (Figure 6b). The parsimony analysis with all taxa and characters also recovered the same *Dinodontosaurus* composition and relationships among the components of this genus, that is, MCP‐1645‐PV as sister to *Dinodontosaurus brevirostris* (Figure S1). The Bayesian inference performed on the dataset with all taxa recovered the same relationship between the members of the *Dinodontosaurus* genus as did the results of the analysis on the reduced dataset (Figures S2–S3). For node support values (Bremer and posterior probabilities, for Parsimony and Bayesian analyses, respectively), and the list of synapomorphies, please see Data S2.

## 4 DISCUSSION

The specimen MCP‐1645‐PV was initially assigned to *Chanaria* sp. by Araújo (1981). However, the cranial morphology, upon detailed analysis, indicates its attribution to the genus *Dinodontosaurus* as currently defined by Kammerer and Ordoñez (2021). MCP‐1645‐PV has all the characteristics listed by those authors in the diagnosis of the genus: presence of tusks, notably elongated and curved; suborbital process of the postorbital bone; a well‐developed anterior process of the frontals; and the demarcation between the caniniform and zygomatic processes of the maxilla. The assignment of MCP‐1645‐PV to *Dinodontosaurus brevirostris* is supported by the presence of a triangular intertemporal bar, differing from *Dinodontosaurus tener*, in which it is uniformly wide along its entire length or at least symmetrically expanded in its anterior and posterior ends (Kammerer & Ordoñez, 2021). Also, Cox (1965, 1968), and Kammerer and Ordoñez (2021) discussed several other characteristics that differentiate *Dinodontosaurus brevirostris* from *Dinodontosaurus tener*, all of them observed in MCP‐1645‐PV. For instance, the postorbital bone makes a posteromedial contact with the parietal along nearly half the length of the intertemporal bar, as observed in *Dinodontosaurus brevirostris* (e.g., the holotype PULR‐V 15; see also Cox, 1968). In contrast, in *Dinodontosaurus tener*, the posterior processes of the postorbitals are restricted to the first third of the intertemporal bar (Cox, 1965). Additionally, according to Kammerer and Ordoñez (2021), the position of the pineal foramen also varies between the two species. In *Dinodontosaurus tener*, the pineal foramen is positioned at the transition between the intertemporal bar and interorbital skull roof, level with the postorbital bars. In *Dinodontosaurus brevirostris*, however, the foramen is situated more posteriorly, in the intertemporal bar sensu stricto, a condition also observed in MCP‐1645‐PV. Finally, another feature that varies between the two *Dinodontosaurus* species is the angle formed between the zygomatic and temporal branches of the squamosal, which also determines the overall shape of the temporal fenestra. In *Dinodontosaurus tener*, this angle is noticeably less than 90°, resulting in a posterolaterally extended fenestra. Conversely, in *Dinodontosaurus brevirostris* (including MCP‐1645‐PV), the branches are arranged in such a way that the angle is nearly right, giving the temporal fenestra a more quadrangular or circular aspect in the dorsal view.

The results presented here suggest intriguing patterns of morphological variation in *Dinodontosaurus tener* and *Dinodontosaurus brevirostris*, both when analyzed separately and in combination (see Supporting Information figures). The relative length of the preorbital region exhibits an asymmetric distribution with a long tail towards larger values in the combined sample, suggesting that most individuals have moderate proportions for this metric. This general pattern, however, may obscure interspecific differences that have been observed in separate analyses. Indeed, when considering the species individually, *Dinodontosaurus tener* is concentrated in lower values of this metric, whereas *Dinodontosaurus brevirostris* dominates intermediate and high values. The relative width of the rostrum in the combined sample exhibits a subtle bimodal pattern, indicating the possible existence of anatomical subgroups or extensive variation within the sample. However, the statistical tests we have run show no support for bimodality for any of the proportions investigated (see Table 2).

When separated by species, this metric reveals that *Dinodontosaurus brevirostris* tends to have higher values, whereas *Dinodontosaurus tener* is concentrated around median values and exhibits greater dispersion. This difference is consistent with a proportionally broader rostrum in *Dinodontosaurus brevirostris*, possibly related to functional variability, such as feeding strategies or ecological adaptations. The proportion between rostrum length and width presents significant overlap between the species, with *Dinodontosaurus tener* displaying slightly higher values. In the combined sample, the distribution is dispersed but with a peak at intermediate values. This metric appears to be less informative for distinguishing the species, although it still reflects significant morphological variation within the group. On the other hand, the relative length of the fenestra exhibits distinct patterns indicative of functional or phylogenetic variation. While *Dinodontosaurus brevirostris* is concentrated in intermediate values, *Dinodontosaurus tener* shows higher density at both low and high values. Notably, the distribution of the relative fenestra length exhibits two coincident peaks for both species (Figure 5d). This may suggest a biological, rather than taphonomic, cause, such as sexual dimorphism. However, regarding this proportion, when the tests were run for each species, no evidence for bimodality was again found. Finally, it is observed that, compared to *Dinodontosaurus brevirostris*, the materials attributed to *Dinodontosaurus tener* tend to exhibit greater variation in the studied cranial proportions, which may be related to taphonomic factors associated with the analyzed specimens.

Fossil specimens recovered from the *Dinodontosaurus* Assemblage Zone, such as MCP‐1645‐PV, tend to exhibit diagenetic modifications resulting from disruptive calcite recrystallization during eodiagenesis (Holz & Schultz, 1998). Nevertheless, MCP‐1645‐PV does not appear to have undergone significant modifications in this regard, lacking the characteristic “swelling” easily detectable in specimens where calcite recrystallization has acted more severely. It is also worth noting that the anatomical parameters used to characterize MCP‐1645‐PV as *Dinodontosaurus brevirostris* are independent of taphonomy (e.g., the pattern of contact between bone sutures).

In addition to Araújo (1981), other researchers have attributed or tentatively suggested the attribution of Brazilian material to the genus “*Chanaria*” (currently considered a synonym of *Dinodontosaurus*) or even *Dinodontosaurus brevirostris*. In Morato's (2006) unpublished dissertation on *Dinodontosaurus*, there is a passage that reviews the taxonomic challenges in distinguishing species of tusked dicynodonts from the Triassic of Brazil, focusing on the unpublished work of Machado (1992). Using canonical linear discriminant analysis, Machado (1992) attempted to distinguish between species such as *Chanaria* sp., *Dinodontosaurus brevirostris*, and *D. turpior* based on diagnostic cranial features. He identified distinct morphometric groupings, especially concerning skull proportions and the size of the temporal fenestra, rostrum, and cranial height. Despite this, Machado (1992) acknowledged that *Dinodontosaurus brevirostris* and *Chanaria* sp. are morphologically very similar (to which our findings agree), with their measurements falling within the variation range of *D. turpior*. Some specimens assigned to *D. turpior* were even more similar to the *Dinodontosaurus brevirostris* group, suggesting possible misclassification. This result is in agreement with what we have shown: these proportions are not reliable.

One of the specimens Machado (1992) used in his analysis is MCP‐0648‐PV from Dona Francisca municipality (Brazil), which he considered as *Chanaria*. We have also examined this specimen, which shows a parietal triangle immediately posterior to the pineal foramen. However, the triangle is relatively small, as the parietals converge rapidly and continue posteriorly as a single, fused structure. This configuration contrasts with other specimens in which the parietals diverge for a longer distance, resulting in a more prominent triangular shape. However, it is similar to what is seen in Argentinian specimens of *Dinodontosaurus brevirostris* (e.g., PULR‐V 15 and MCZ VPRA‐3543; see Kammerer & Ordoñez, 2021, fig. 24). One aspect different from the *Dinodontosaurus brevirostris* morphology is that the postorbitals of MCP‐0648‐PV do not extend beyond the pineal foramen. We have thus considered this specimen as *Dinodontosaurus tener*. It is important to note that the specimen has undergone some reconstruction, particularly on the right side of the skull. While the medial part of the occipital region appears to be original, the tusks are entirely reconstructed, and the left caniniform process has been polished, possibly obscuring original surface details. Additionally, the specimen is dorsoventrally compressed. These factors lead to caution in the interpretation of some aspects of MCP‐0648‐PV morphology.

Schwanke and Melo (2002) briefly described MCT.R.1478, a juvenile dicynodont specimen from the Pinheiro region, Candelária municipality, Brazil. They note some similarities between the specimen they described and *Chanaria*; for example: “A character similar to *C. platyceps* is found in the posterior expansion of the postorbital, which extends to the intertemporal bar, well beyond the pineal foramen” (Schwanke & Melo, 2002, p. 182). A considerable extension of the posterior process of the postorbitals in the intertemporal region is a feature we have also observed in MCP‐1645‐PV. In *Dinodontosaurus tener* specimens, the postorbitals are more anteriorly restricted. Schwanke and Melo (2002), however, did not consider *Chanaria* as sufficiently distinct from *Dinodontosaurus*. These authors also emphasized that, given the ontogenetically immature state of the specimen, they could not refer with enough certainty to either *Dinodontosaurus* or *Chanaria*. In addition to the morphology of the postorbital, we also point out that posteriorly to the pineal foramen, the intertemporal bar of MCT.R.1478 forms a structure similar to the triangle seen in *Dinodontosaurus brevirostris* adult specimens (e.g., CRILAR‐Pv 94, PULR‐V 15, PULR‐V 144; see also Kammerer & Ordoñez, 2021, fig. 24; Escobar et al., 2023, fig. 1).

Other Brazilian specimens from the Sanga Janguta outcrop, Candelária municipality, may be referable to *Dinodontosaurus brevirostris*. The specimen MCT.R.213 was depicted in Cox (1968, fig. 5f) and attributed to “*D. turpior*.” In this figure, all specimens he refers to as “*D. turpior*” show postorbitals restricted to the pineal foramen, while in MCT.R.213 the postorbitals extend far back, as seen in *Dinodontosaurus brevirostris* specimens. Another specimen, MCT.R.378 from a nearby site the “Sanga do Pinheiro,” bears postorbitals that extend further back on the skull, beyond the pineal foramen. Additionally, the temporal fenestra is almost quadrangular, as in *Dinodontosaurus brevirostris* specimens. This specimen, however, does not represent an adult individual (basal skull length ~22 cm). It is interesting to note that the resemblance of MCT.R.213 (and other specimens from the MCT collection) with *Dinodontosaurus brevirostris* had already been noted by Cox (1968). However, this author discarded its taxonomic value and referred to this material as “*D*. *turpior*” without further discussion, explaining the differences in the intertemporal morphologies as intraspecific variation.

The identification of *Dinodontosaurus brevirostris* among the few tusked dicynodont specimens recovered from the Vale Verde region is particularly intriguing, given the limited knowledge about the fossil diversity of this area. The paleontological sites of Vale Verde comprise a series of historical outcrops associated with a railroad cut, in which the precise location of just one point is known nowadays: the Cria Farm Site. This farm is mentioned in catalogue books, but there is a possibility that other historical outcrops were also explored and labeled as “Vila Melos” or “General Camara,” which were previous names of the Vale Verde municipality and may have also been also used for the outcrops of the Cria Farm (see Martinelli et al., 2017). Aside from this historical problem, other *Dinodontosaurus* sp. specimens (e.g., MCP‐0890‐PV, MCP‐1362‐PV, and MCP‐1602‐PV) were also found in the area, although their preservation precluded us from identifying them at the species level. The type material of the chiniquodontid cynodont *Aleodon cromptoni* (Martinelli et al., 2017) comes from the “Cria Farm Site,” and other herbivorous traversodontid cynodonts are also known for the Vale Verde fauna, like the genera *Massetognathus* and *Luangwa* (Martinelli et al., 2017). Archosauromorphs such as carnivorous pseudosuchians (Schultz et al., 2020) and possible rhynchosaur materials (VDPN, personal communication) are also known.

This diversity context and the results of the present study confirm a typical community of the *Dinodontosaurus* Assemblage Zone (DAZ) for the Vale Verde region. This faunal biozone is one of the most diverse in the Brazilian Triassic (Schultz et al., 2020), and the recognition of *Dinodontosaurus brevirostris* further increases its paleodiversity. However, Martinelli et al. (2017) argue that DAZ may be divisible into two distinct biozones, with *Aleodon*, *Luangwa*, and *Brasinorhynchus* as putative older components in this division—two of which are taxa found in the Vale Verde region. It is worth noting that this assemblage (*Aleodon*, *Luangwa*, and *Massetognathus*) is unusual compared to the “typical” DAZ fauna, as these genera are originally (and more abundantly) known from Tanzania, Zambia, and Argentina, respectively. This pattern suggests that the Vale Verde region may represent a temporally or environmentally distinct setting compared to other Brazilian DAZ localities and possibly indicates faunal links with the Los Chañares Formation. Thus, the presence of *Dinodontosaurus brevirostris* in this region may play an important role in future biostratigraphical discussions regarding this disruptive scenario. Most likely, a sympatry of *Dinodontosaurus* species may be expected since other *Aleodon*‐and‐*Luangwa* outcrops also present putative records of *Dinodontosaurus tener*.

The results of this research have important biostratigraphic implications. *Dinodontosaurus* has been one of the main elements of correlation between the DAZ and the Chañares Formation of Argentina, specifically the *Massetognathus*‐*Chanaresuchus* Assemblage Zone (MCAZ) (Ezcurra et al., 2017; Martinelli et al., 2024). The shared presence of this taxa has been generally established at a genus‐level range, with endemic species in Argentina and Brazil. Exceptions include two previous proposals: (a) *Dinodontosaurus* as a monospecific genus for both sequences (Lucas & Harris, 1996; Langer et al., 2007), and (b) the few cases where taxa typical of the MCAZ (i.e., *Chanaria* and *Dinodontosaurus brevirostris*) were also reported in the DAZ (Araújo, 1981; Machado, 1992). The present contribution is consistent with proposal (b), with *Dinodontosaurus brevirostris* as a common species for the MCAZ of the Chañares Formation and the DAZ of the Pinheiros‐Chiniquá Sequence.

## 5 CONCLUSION

The present reassessment of the specimen MCP‐1645‐PV, previously assigned to *Chanaria* sp. (currently considered a synonym of *Dinodontosaurus brevirostris*; Kammerer & Ordoñez, 2021), significantly contributes to the understanding of morphological variability within the genus *Dinodontosaurus*, particularly in the taxonomic attribution of its species. The morphological reevaluation of the specimen's skull was conducted using modern methods such as photogrammetry and statistical analyses, providing new insights into previously established diagnostic characteristics.

Our reassessment of MCP‐1645‐PV is consistent with the proposal by Araújo (1981), with observed features supporting its attribution to *Dinodontosaurus brevirostris*, including the subtriangular shape of the intertemporal bar, a quadrangular shape of the temporal fenestra in dorsal view, and postorbitals that extend further posteriorly along the intertemporal bar.

Regarding the measured proportions, the results warrant caution in their use for assigning specimens to different *Dinodontosaurus* species. The analysis of these measurements reveals considerable intraspecific variation, particularly in *Dinodontosaurus tener*, while interspecific variation is not distributed in a manner that allows for a clear separation between the two species.

The recognition of *Dinodontosaurus brevirostris* in the *Dinodontosaurus* Assemblage Zone challenges the traditional hypothesis of taxonomic homogeneity of the non‐stahleckeriine dicynodonts from this association. This demonstrates that the Kannemeyeriiformes diversity in the Ladinian‐Carnian of South America is an open issue, which we are still far from fully understanding. A comprehensive anatomical and taxonomic revision of *Dinodontosaurus* (in the case of *Dinodontosaurus brevirostris* JAE has a work in progress) will probably shed additional light on this topic.

## AUTHOR CONTRIBUTIONS

**Julia Lara Rodrigues de Souza:** Conceptualization; investigation; writing – original draft; methodology; visualization; writing – review and editing; software; formal analysis; data curation; project administration; validation. **João Lucas Da Silva:** Conceptualization; investigation; writing – original draft; methodology; validation; writing – review and editing; software; formal analysis; data curation; visualization. **Voltaire D. P. Neto:** Validation; visualization; writing – review and editing. **Arielli Fabrício Machado:** Validation; writing – review and editing; software. **Juan A. Escobar:** Validation; writing – review and editing; formal analysis; investigation. **Felipe L. Pinheiro:** Funding acquisition; writing – original draft; methodology; validation; visualization; writing – review and editing; formal analysis; supervision; resources; project administration.

## Supporting information


**Data S1.** Supporting Information.

**Data S2.** Supporting Information.

## Data Availability

Data for phylogenetic and other statistical analyses performed here, as well as Mrbayes and R scripts, are available at Morphobank: http://morphobank.org/permalink/?P5978.
